# The Confinement-Affected Strength Variety of Anisotropic Rock Mass

**DOI:** 10.3390/ma15238444

**Published:** 2022-11-27

**Authors:** Songfeng Guo, Shengwen Qi, Bowen Zheng, Lei Xue, Xueliang Wang, Ning Liang, Yu Zou, Fengjiao Tang, Waqar Muhammad Faisal, Weiluan Wen, Yongchao Li, Xin Yu

**Affiliations:** 1 Key Laboratory of Shale Gas and Geoengineering, Institute of Geology and Geophysics, Chinese Academy of Sciences, Beijing 100029, China; 2 Innovation Academy for Earth Science, Chinese Academy of Sciences, Beijing 100029, China; 3 University of Chinese Academy of Sciences, Beijing 100049, China

**Keywords:** rock mass, anisotropy, confining pressure, strength, failure mechanism, discontinuity

## Abstract

It has been recognized that the anisotropic structures dominate the deformation and strength properties of laminated rock masses. The resultant strength anisotropy is strongly affected by confining pressures beyond anisotropic structures. Nevertheless, the effects of confinement are inconsistent among existing experiments and not fully understood. This study focuses on the effects of confining pressure on strength anisotropy through theoretical derivation together with experimental results analysis. The variations in the possibility of anisotropic structural plane dominant failure and strength anisotropy degree under different confining pressures are discussed. The different types of anisotropic structural planes, i.e., the fresh contact discontinuity or soft, thick layer, are found as the key factor resulting in different confinement effects. The strength anisotropy weakens gradually and vanishes eventually as confining stress increases for the anisotropic rock mass with the structural plane of fresh contact discontinuity. On the other hand, the strength does not vanish at very high confining stress and the anisotropic strength difference even rises as confining stress increases for the anisotropic rock mass with the anisotropic structural plane of the soft layer. This study improves the understanding of anisotropic rock mass mechanical behavior, especially at high confining stress, and may promote the development of excavation and supporting techniques for underground projects.

## 1. Introduction

It is commonly believed that rock mass is heterogeneous and discontinuous, containing fissures, joints, faults, cleavage planes, and bedding planes and these structural planes dominate the mechanical behaviors of rock mass [1,2,3,4,5,6,7]. Different loading directions concerning structural planes orientations always make rock mass anisotropic and more problematic during engineering construction [8,9,10,11,12,13,14,15,16,17]. 

**Figure 1:**
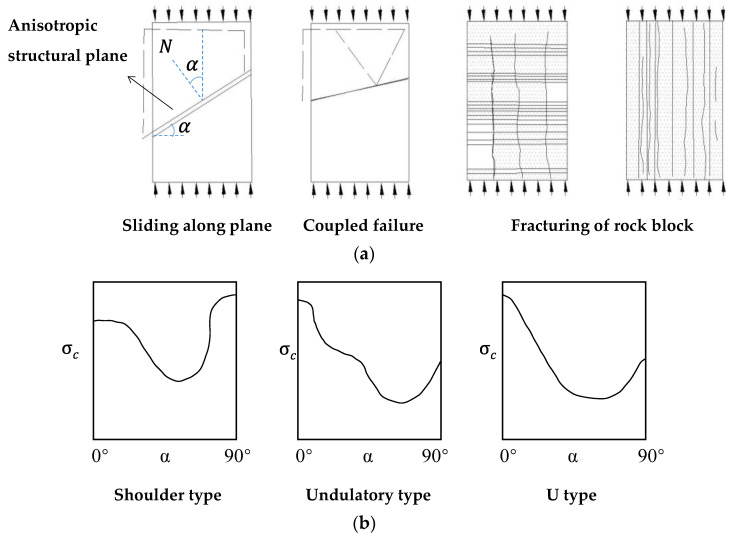
The direction dominant mechanical behavior of anisotropic rock mass. (**a**) The failure types anisotropy; (**b**) the strength anisotropy (modified based on [35]).

Numerous types of rock mass have inherent or structural anisotropy, such as parametamorphic and sedimentary rocks, and other discontinuity-induced stratiform-like rock masses. Extensive compression tests have been put forward to explore the strength and failure features of various anisotropic rock masses at uniaxial and triaxial compression conditions in the laboratory, e.g., slate [18,19,20], schist [13,20,21,22,23,24], phyllite [13,25], marble [13,23], sandstone [3], shale [26,27,28], limestone [28,29,30], mudrock [31], columnar basalt [32], and artificially anisotropic rock specimens [3,33]. Numerical studies have also been conducted to investigate the mechanical behavior of anisotropic rock mass intrinsically [12,34]. These effective studies reflect that the deformation and strength properties are largely dominated by the angle between the normal anisotropic structural plane and the direction of minimum principal stress (α). As shown in Figure 1, most of the anisotropic rock mass had maximum strength at a critical angle around α=0° or 90° and failed as the rock block fractured, while they had a minimum around α = 45° + *φ*_*an*_/2 and failed while sliding along the anisotropic structural plane, where $\varphi_{an}$ is the friction angle of anisotropic structural plane.

As the depth of engineering construction tends to be greater and greater in recent years, the environmental geostress of rock mass reaches as high as tens of MPa. The mechanical properties of anisotropic rock mass under high confining stress are more concerning among rock mass geomechanical researchers. The existing research has indicated that the confining stress greatly affects the strength anisotropy by contributing to the normal stress on the weak plane [24,36,37,38]. Sun (1988) noted that although the rock mass structure dominates the mechanical behavior of the rock mass at low confining stress state, the domination of the rock mass structure is restricted as confining stress increases [2]. His viewpoint was supported by some triaxial compression tests, which showed that the strength anisotropy decreased gradually with confining pressure increases and finally disappeared at high enough confining pressure [38,39]. The transformation critical confining stress of rock mass strength from anisotropy to isotropy was proposed and comparable with the experiments [40]. The recently developed classification methods for anisotropic rock mass consider the weaken effects of confining pressure on the anisotropy degree [41,42]. However, some experiments also indicate that strength anisotropy exists even at very high confining pressure, e.g., dolomitic limestone has strength anisotropy at a confining stress of 80 MPa [29]. Thus, the strength anisotropy properties of rock mass under various confining pressure are not fully understood and need further discussions. 

This study focuses on the effects of confining pressure on strength anisotropy through theoretical derivation along with experimental results analysis. The variations in the possibility of anisotropic structural plane dominant failure and strength anisotropy degree under different confining pressures are discussed. The different types of anisotropic structural plane are the key factors resulting in different confinement effects.

## 2. Theoretical Analysis on Strength of Anisotropic Rock Mass

**Figure 2:**
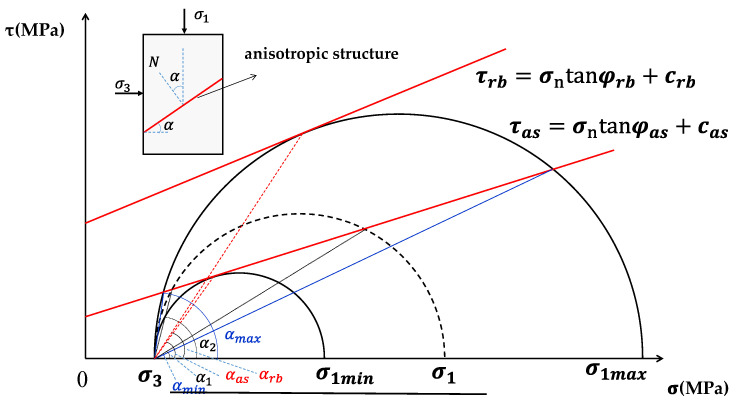
The Mohr circle analysis on limit failure condition of anisotropic rock mass. and $\tau_{as}$ denote the shear strength of rock block and anisotropic structural plane, respectively, while $\sigma_{n}$ denotes the normal stress on shear failure plane.

Several researchers have developed strength prediction methods for anisotropic rock mass in order to depict the strength variations with the orientation of anisotropic structural plane under different confining stresses. These methods are mostly modified based on Griffith theory [43], Mohr–Coulomb strength criterion [1,28,36,44,45,46,47,48,49,50,51,52], Hoek–Brown strength criterion [23,41,53,54], and other empirical formulas [19,22,25,55] or the fuzzy method [21,56]. Among these methods, the Mohr–Coulomb strength criterion based on the single plane of weakness theory [1] is one of the most widely used. The anisotropic structural plane is a well-defined parallel discontinuity, and the rock block is regarded as isotropic, both are depicted by Mohr–Coulomb criterion in the classic Jaeger’s criterion. In this study, the widely used Mohr–Coulomb strength threshold is adopted to depict the strength of rock block and anisotropic structural plane. The strength and failure mode of an anisotropic rock specimen under confining pressure ($\sigma_{3}$) are exhibited with Mohr cycles in Figure 2. The strength of anisotropic rock specimen changes with the inclined angle α of anisotropic structural plane. The potential maximum and minimum strengths can be signified as Equations (1) and (2).

(1) $$\sigma_{1max}=\frac{1+\sin\varphi_{rb}}{1-\sin\varphi_{rb}}\sigma_{3}+\frac{2c_{rb}\cos\varphi_{rb}}{1-\sin\varphi_{rb}}\;\;\;\;\;\mathrm{when}\;\alpha =45^{{}^{\circ}}+\frac{1}{2}\varphi_{rb}$$

(2) $$\sigma_{1min}=\frac{1+\sin\varphi_{as}}{1-\sin\varphi_{as}}\sigma_{3}+\frac{2c_{as}\cos\varphi_{as}}{1-\sin\varphi_{as}}\;\;\;\;\;\mathrm{when}\;\alpha =45^{{}^{\circ}}+\frac{1}{2}\varphi_{as}$$

where $\sigma_{1max}$ and $\sigma_{1min}$ denote the potential maximum and minimum strength of anisotropic rock specimen strength, respectively. $\varphi_{rb}$ and $c_{rb}$ denote the internal friction angle and cohesion of the rock block, respectively; while $\varphi_{as}$ and $c_{as}$ denote the friction angle and cohesion of the anisotropic structural plane, respectively. 

As shown in Figure 2, the failure of anisotropic rock mass should be sheared through the rock block when $\alpha <\alpha_{min}$ or $\alpha >\alpha_{max}$ and its strength can be presented by Equation (1). On the other hand, the failure of anisotropic rock mass is most likely to slide or shear along the anisotropic structural plane when $\alpha_{min}<\alpha <\alpha_{max}$, and its strength is between the results reached by Equations (1) and (2), which is shown in Equation (3).

(3) $$\sigma_{1as}=\sigma_{3}\tan\alpha \cot\left(\alpha -\varphi_{as}\right)+\frac{c_{as}}{\cos\alpha \left(\sin\alpha -\cos\alpha \tan\varphi_{as}\right)}\;\;\;\;\;\mathrm{when}\;\alpha_{\min}<\alpha <\alpha_{\max}$$

where $\sigma_{1as}$ denotes the strength of anisotropic rock mass, $\alpha_{max}$ and $\alpha_{min}$ denote the maximum and minimum inclined angle α between which anisotropic rock mass strength is dominant by anisotropic structural plane.

This indicates that the strength relates closely to the applied stress direction α in Equation (3). The minimum strength expressed in Equation (2) is a special case of Equation (3) at $\alpha =45^{\circ }+\frac{1}{2}\varphi_{\mathrm{as}}$. In addition, the strength reached in Equation (3) is equivalent to that reached in Equation (1) when $\alpha =\alpha_{min}$ or $\alpha =\alpha_{max}$, and the anisotropic structural plane and rock block dominate the failure mechanism cooperatively. The two-angle thresholds $\alpha_{min}$ and $\alpha_{max}$ can be obtained based on the equivalence of Equations (1) and (3), and the Mohr circle analysis, see Equations (4) and (5).

(4) $$\alpha_{min}=\frac{1}{2}\sin^{-1}\left\{\left[1+\frac{\left(c_{as}\cot\varphi_{as}+\sigma_{3}\right)\left(1-\sin\varphi_{rb}\right)}{\sigma_{3}\sin\varphi_{rb}+c_{rb}\cos\varphi_{rb}}\right]\sin\varphi_{as}\right\}+\frac{1}{2}\varphi_{as}$$

(5) $$\alpha_{max}=\frac{\pi }{2}+\varphi_{as}-\alpha_{min}$$

This indicates from the equations that the critical angles $\alpha_{min}$ and $\alpha_{max}$ are not only the functions of strength parameters but also for confining stress.

## 3. Verification Study of Existing Experimental Data

**Figure 3:**
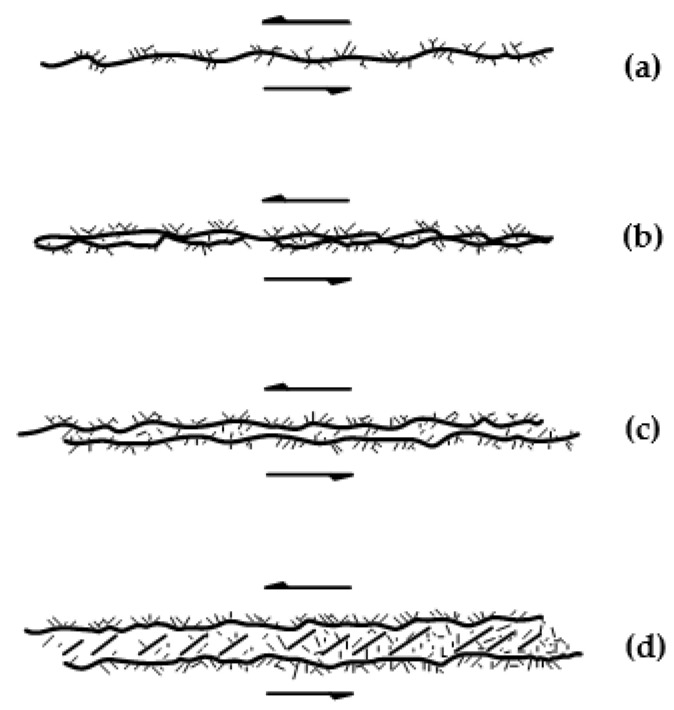
The types of discontinuity. The fresh rough contact joint is well-exhibited in (**a**), while the filled layer becomes thicker and thicker (**b**–**d**) denoting the soft discontinuous layer.

The structural planes that influence the anisotropic property can be generally grouped into two types according to mechanical properties, i.e., weak and hard discontinuities (Figure 3). The former includes bedding planes in sedimentary rocks, weak intercalated layers in rock matrix. The latter mainly refers to fresh and clean fractures (or relatively stiff interlayers sometimes) within the rock matrix. In this section, the experimental results involving different types of discontinuities in literatures are presented to assess the availability of above analysis. The dolomitic limestone in Section 3.1 and sandstone in Section 3.2 are sedimentary rocks and thus their anisotropic structural planes are mainly bedding planes regarded as weak planes. Comparatively, the anisotropic structural planes of plaster of Paris in Section 3.3 are an artificial contact joint, regarded as hard discontinuities.

### 3.1. Dolomitic Limestone

**Figure 4:**
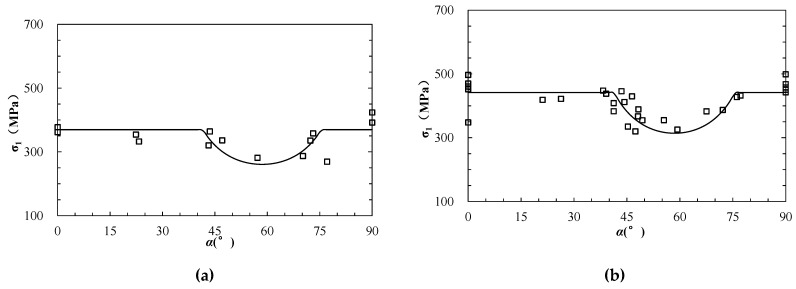

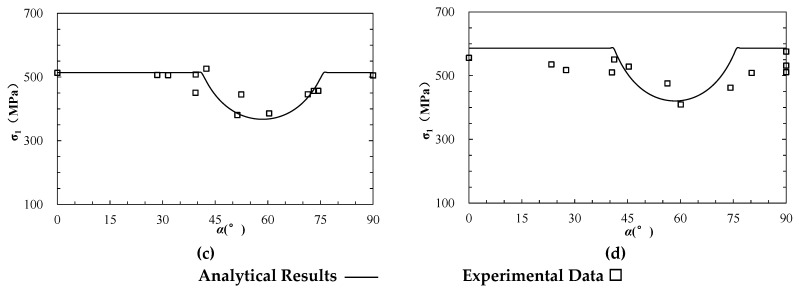
The analytical and experimental strength of anisotropic dolomitic limestone (modified based on [34]). (**a**–**d**) represent the results under triaxial compression at different confining pressure ($\sigma_{3}$) of 20, 40, 60, 80 MPa, respectively.

A large number of experimental studies on the strength of layered rock mass were conducted under varied confining pressures [29]. The dolomitic limestone of Manlius formation was prepared as a cylindrical rock specimen with a length of 10 cm and diameter of 1.2 cm. The tests were carried out under four levels of confining pressures of 20 MPa, 40 MPa, 60 MPa, and 80 MPa, respectively, and the inclined angles of layers (α) ranged from 0° to 90°. The internal friction angle ($\varphi_{rb}$) and cohesion ($c_{rb}$) of the matrix (rock block) were 34.5° and 78.23 MPa, respectively, while the internal friction angle ($\varphi_{as}$) and cohesion ($c_{as}$) of limestone layer (anisotropic structural plane) were 27° and 63.56 MPa, respectively (Table 1). It should be noted that the specimen size is much smaller than the standard specimen with diameter of 5 cm and length of 10 cm at least, and as the size effect the strengths are much higher than those reported in other literatures, e.g., 15.765–124.74 MPa in [57]. The experimental results and the estimated values based on Equations (1)–(3) are presented in Figure 4 as hollow squares and lines, respectively. It can be seen that the estimated strengths are generally comparable with the experimental results. 

### 3.2. Lyonian Sandstone 

**Figure 5:**
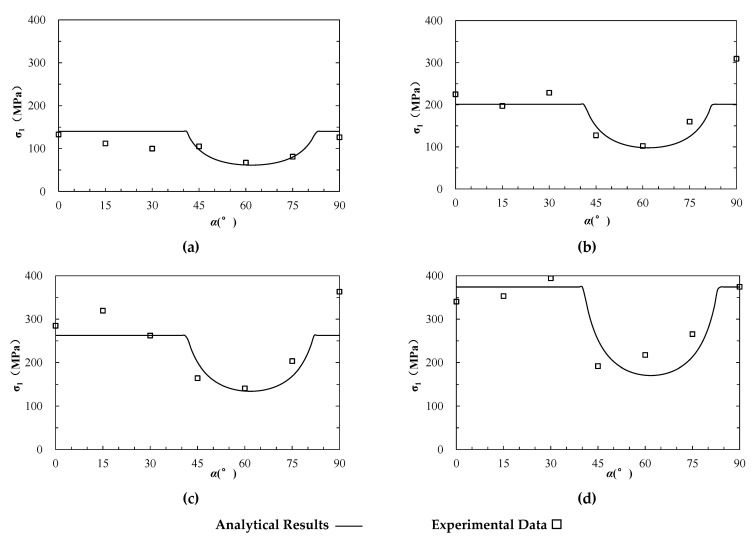
The analytical and experimental strength of anisotropic sandstone (modified based on [58]). (**a**–**d**) represent the results under uniaxial compression and triaxial compression at different confining pressure ($\sigma_{3}$) of 10.5, 21 and 31.5 MPa, respectively.

Sandstone is a typical anisotropic rock stem from a bedding plane formed during the depositional process. A series of triaxial compression tests were performed on Lyonian sandstone in different directions [58]. The specimens were prepared as cylindrical shapes with height of 10.8 cm and a diameter of 5.4 cm. The inclined angle of the bedding plane ranged from 0° to 90°, and confining pressures incorporated four levels of 0, 10.5 MPa, 21 MPa, and 31.5 MPa. The internal friction angle ($\varphi_{rb}$) and cohesion ($c_{rb}$) of the rock block were 47.92° and 26.92 MPa, respectively; while the internal friction angle ($\varphi_{as}$) and cohesion ($c_{as}$) of the anisotropic structural plane were 33.5° and 16.45 MPa, respectively (Table 1). The experimental results and the estimated values based on Equations (1)–(3) are presented in Figure 5 as hollow squares and lines, respectively, which are compared with each other.

### 3.3. Plaster of Paris with Artificial Contact Joint

**Figure 6:**
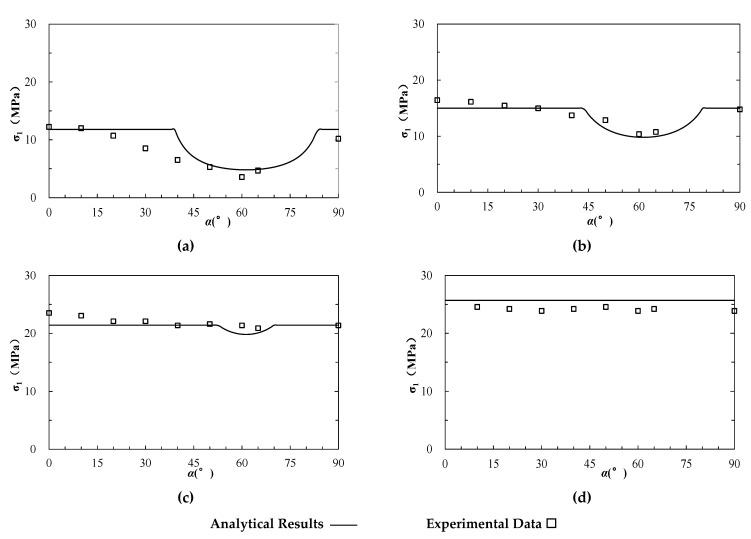
The analytical and experimental strength of anisotropic jointed plaster of Paris (modified based on [3]). (**a**–**d**) represent the results under triaxial compression at different confining pressure ($\sigma_{3}$) of 0.5, 2, 5, and 7 MPa, respectively.

Ramamurthy and Arora (1994) conducted confined compression tests on the plaster of Paris with artificial contact discontinuities [3]. The specimens were prepared with a diameter of 38 mm and height of 76 mm. A number of cleanly and roughly broken joints at various inclinations (α = 0°, 10°, 20°, 30°, 40°, 50°, 60°, and 90°) were developed by breaking the specimens in the direction of the prenotch. Different confining pressures of 0.3 MPa, 0.5 MPa, 1 MPa, 1.5 MPa, 2 MPa, 5 MPa, and 7 MPa were applied. The cohesion and internal friction angle of plaster of Paris are 3.67 MPa and 21.26°, respectively. Comparatively, the cohesion and frictional angle of discontinuities are about 0.86 MPa and 32.56°, respectively (Table 1). The estimated strength based on Equations (1)–(5) was presented at different confining pressures as well as the corresponding experimental data (Figure 6). The results show an acceptable fit between the estimated and real data at different confining levels. 

## 4. Discussion

### 4.1. The Effects of Confinement on the Possibility of Anisotropic Structural Plane-Controlled Strength

The scope of incline angle α falling between $\alpha_{min}$ and $\alpha_{max}$ can indicate the possibility of anisotropic structural plane-controlled failure, which shows different trends as confining stress increases in various cases, e.g., the data in Figure 4, Figure 5 and Figure 6. The causing factors needs a thorough discussion. If using *A*($\sigma_{3}$) substitutes for the formula for confining stress ($\sigma_{3}$) in Equation (4), we can reach the derivative of *A*($\sigma_{3}$) as follows.

(6) $$\begin{array}{c} \frac{\partial A\left(\sigma_{3}\right)}{\partial \sigma_{3}}=\frac{\partial \left[1+\frac{\left(c_{as}\cot\varphi_{as}+\sigma_{3}\right)\left(1-\sin\varphi_{rb}\right)}{\sigma_{3}\sin\varphi_{rb}+c_{rb}\cos\varphi_{rb}}\right]}{\partial \sigma_{3}}\\ =\frac{\left(c_{rb}\cot\varphi_{rb}-c_{as}\cot\varphi_{as}\right)\left(1-\sin\varphi_{rb}\right)\sin\varphi_{rb}}{{\left(\sigma_{3}\sin\varphi_{rb}+c_{rb}\cos\varphi_{rb}\right)}^{2}} \end{array}$$

The equation indicates that the positive or negative is dominated by the relationship between $c_{rb}\cot\varphi_{rb}$ and $c_{as}\cot\varphi_{as}$ that signifies the values of the intersection of the shear strength and lateral axis for rock block and anisotropic structural plane, respectively, shown in Figure 2.

① When $c_{rb}\cot\varphi_{rb}>c_{as}\cot\varphi_{as}$, $\frac{\partial A\left(\sigma_{3}\right)}{\partial \sigma_{3}}$is positive, which means that $A\left(\sigma_{3}\right)$ increases with $\sigma_{3}$. This signifies that $A\left(\sigma_{3}\right)$ (or $\alpha_{min}$) obtains its minimum value at $\sigma_{3}=0$. The scope of $\alpha_{min}<\alpha <\alpha_{max}$ narrow down as confining stress ($\sigma_{3}$) increases, which means the confinement reduces the anisotropic structural plane failure possibilities under this condition.

② When $c_{rb}\cot\varphi_{rb}<c_{as}\cot\varphi_{as}$, $\frac{\partial A\left(\sigma_{3}\right)}{\partial \sigma_{3}}$ is negative, which means that $A\left(\sigma_{3}\right)$ decreases with $\sigma_{3}$. This signifies $A\left(\sigma_{3}\right)$ (or $\alpha_{min}$) obtains its maximum value at $\sigma_{3}=0$. The scope of $\alpha_{min}<\alpha <\alpha_{max}$ expands as confining stress ($\sigma_{3}$) increases, which means that the confinement increases the anisotropic structural plane failure possibilities under this condition.

For the three experimental cases presented above, the corresponding $c_{rb}\cot\varphi_{rb}$ and $c_{as}\cot\varphi_{as}$ are shown in Table 1. The results indicate that $c_{rb}\cot\varphi_{rb}$ is the relatively small one for dolomitic limestone, while the relatively big one is for sandstone and jointed plaster of Paris. Thus, the possibility of an anisotropic structural plane-controlled failure increases for dolomitic limestone and decreases for sandstone and jointed plaster of Paris as confinement increases. 

**Figure 7:**
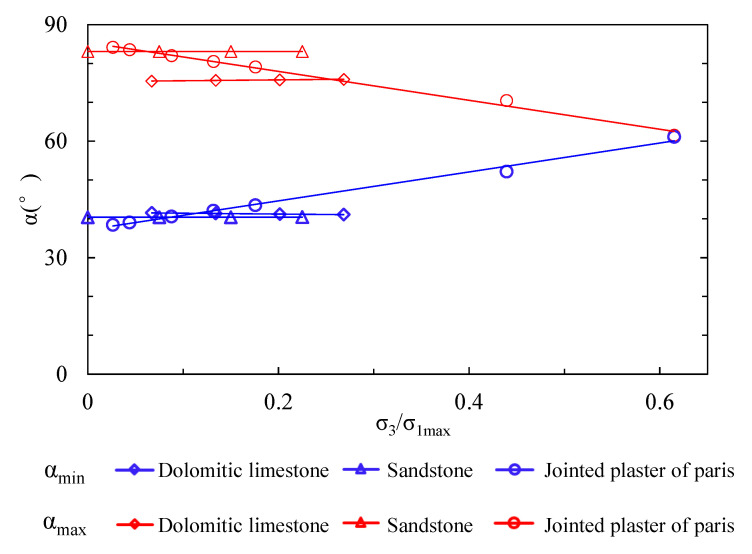
The relation between possibility of anisotropic structural plane-controlled failure and confinement.

We use the ratio of confining pressure $\sigma_{3}$ to maximum potential strength $\sigma_{1max}$ to non-dimensionalize the confinement. The estimated thresholds of anisotropic structural plane inclination ($\alpha_{min}$ and $\alpha_{max}$) with dimensionless confinement are presented in Figure 7. The tendency of $\alpha_{min}$ decreases while $\alpha_{max}$ increases for dolomitic limestone, which means the scope of $\alpha_{min}\sim \alpha_{max}$ expands up as confinement increases. Conversely, the tendency of $\alpha_{min}$ increases while $\alpha_{max}$ decreases for dolomitic limestone and jointed plaster of Paris, which means the scope of $\alpha_{min}\sim \alpha_{max}$ narrows down as confinement increases. The results agree well with the estimation in Table 1. Moreover, the inclination thresholds of dolomitic limestone and sandstone change slightly with confinement; conversely, the jointed plaster of Paris has a much more obvious change tendency. The $\alpha_{min}$ reaches $\alpha_{max}$ for jointed plaster of Paris when $\sigma_{3}/\sigma_{1max}$ exceeds 0.61, which signifies that the possibility of anisotropic structure-controlled strength is zero at high confining stress. This phenomenon may have a relation with the different degree between $c_{rb}\cot\varphi_{rb}$ and $c_{as}\cot\varphi_{as}$ of the three rock types (Table 1).

### 4.2. The Effects of Confinement on Strength Anisotropy Degree

Researchers have proposed several indexes to determine the anisotropy degree of anisotropic rock mass, e.g., the uniaxial compressive strength anisotropy index [35], and the point load strength anisotropy index [59,60]. In this study, we adopt anisotropic strength ratio (ASR), i.e., the ratio of potential maximum to minimum compressive strength, to discuss the effects of confinement on anisotropy degree (Equation (7)).

(7) $$\mathrm{ASR}=\frac{\sigma_{1max}}{\sigma_{1min}}$$

**Figure 8:**
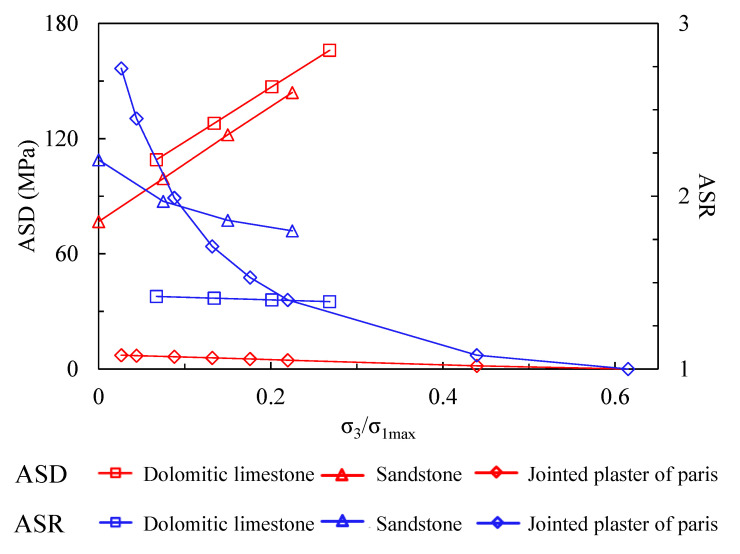
The relation between anisotropy degree of anisotropic rock mass and confinement.

We calculated ASRs of the three rock types under different levels of confinement, and exhibit them as blue lines in Figure 8. The results indicate that ASRs decrease for all three rock types as confinement increases, which means the confinement can weaken the strength anisotropy of rock mass. Among them, the ASR of jointed plaster of Paris tends toward 1 at high confining stresses, possessing strength isotropy. This is in accordance with many existing researches [39,40].

Despite isotropization under high confinement using the index of anisotropic strength ratio, i.e., the absolute value between potential maximum and minimum compressive strength, i.e., the anisotropic strength difference (ASD) (Equation (8)) can reflect whether the rock mass can possess a total strength isotropy when the confinement is high enough.

(8) $$\mathrm{ASD}=\sigma_{1max}-\sigma_{1min}$$

The calculated ASDs are shown as red lines in Figure 8. This indicates that ASD increases for dolomitic limestone and sandstone as confinement increases, and decreases for jointed plaster of Paris. This phenomenon signifies that although the tendency of ASD predicts an anisotropy weakening for dolomitic limestone and sandstone, the potential minimum strength never reaches the maximum strength, and the absolute difference becomes even larger as confinement increases.

The effects of confinement on strength anisotropy are mainly dependent on the formula regarding to the friction strength of rock block and anisotropic structural plane (Equations (1) and (2)). The tendency of ASD with variable confinement can be directly determined by a comparison between $\frac{1+\sin\varphi_{rb}}{1-\sin\varphi_{rb}}$ and $\frac{1+\sin\varphi_{as}}{1-\sin\varphi_{as}}$. ASD increases under the condition of $\varphi_{rb}>\varphi_{as}$, and decreases under the condition of $\varphi_{rb}<\varphi_{as}$. For the exceptional cases of $\varphi_{rb}=\varphi_{as}$, ASD is constant. On the other hand, the factors influencing the ASR tendency are more complicated. 

### 4.3. Further Discussions

The above discussion reflects that the strength anisotropy of an anisotropic rock mass under varied confining pressures is closely related to the strength properties of rock block and anisotropic structural plane. The commonly used Mohr–Coulomb criterion is adopted to depict the strength of rock block and anisotropic structural plane in this study. A number of other strength criteria can also be applied following a similar process, such as the Hoek–Brown strength formula [61]. 

The effects of the anisotropic structural plane on rock mass strength are not always weakened gradually as confining pressure increases, but variable according to the strength properties. This study indicates that the effect of the anisotropic structural plane on rock mass strength does not vanish even under very high confining pressure for the rock mass with relatively soft anisotropic structural planes, e.g., sedimentary rock mass or weak intercalated rock mass (see Figure 3b–d). The strengths of such discontinuities are highly dependent on the thickness and strength of interlayers. On the other hand, the fresh contact discontinuities may lose their effects gradually on the rock mass strength as confinement increases (see Figure 3a). The strength of such discontinuities is dominated mainly by the joint roughness and wall strength [36,62,63,64,65]. Byerlee (1978) performed statistical research on the friction properties of such contact discontinuities. Based on a thorough analysis of rock friction experiments, he concluded that the shear strength of sliding one rock over another varies widely dependent on surface roughness at low normal stress up to 5 MPa [66], and is nearly independent of rock type and surface roughness at high normal stress. The shear strength equations at different normal stresses are presented as Equations (9)–(11).

(9) $$Τ=\sigma_{n}\tan\left[JRClog_{10}\left(\frac{JCS}{\sigma_{n}}\right)+\varphi_{b}\right]\sigma_{n}\leq 5\;\mathrm{Mpa}$$

(10) $$\tau =0.85\sigma_{n}\;5\;\mathrm{Mpa}<\sigma_{n}\leq 200\;\mathrm{Mpa}$$

(11) $$\tau =0.5+0.6\sigma_{n}\;\;\;\sigma_{n}>200\;\mathrm{Mpa}$$

Where JRC, JCS, and $\varphi_{b}$ denote joint roughness coefficient, joint wall compressive strength and basic internal friction angle, respectively.

According to Byerlee’s formula, we can see that at the depth of traditional civil or mining engineering, the friction angle of fresh discontinuities is around 40° (Equation (10)). This is a referable indicator to estimate whether the anisotropic structural plane holds or loses effects on rock mass strength as confining pressure increases. The anisotropic strength difference (ASD) decreases and anisotropic rock mass tends to show features of isotropy as confining pressure increases when the internal friction angle of rock block is smaller than 40°, and vice versa.

It should be noted that this study focuses on the anisotropic rock mass incorporating one set of joints, and thus the presented formulas have limitations for rock mass anisotropy stemming from two and more sets of joints. 

## 5. Conclusions Remarks

Theoretical analyses were conducted on the strength of anisotropic rock mass based on experimental results under different confining pressures. Some concluding remarks were reached as follows:

(1) The anisotropic structural planes incorporate both weak-filled layers and hard contact discontinuities, which cause strength anisotropy and different failure modes of rock mass at low confining pressures;
(2) The commonly used Mohr–Coulomb strength criterion is adopted to depict the strength of both rock block and anisotropic structural plane, based on which the formulas to estimate anisotropic strength under certain confining pressures are developed. The formulas compare well with the compression experiments data of various anisotropic rock types under different confining pressures;
(3) The possibility of anisotropic structural plane-controlled rock strength as confining pressure increases is not definite but theoretically related to the comparison between $c_{rb}\cot\varphi_{rb}$ and $c_{as}\cot\varphi_{as}$;
(4) Likewise, the tendency of strength anisotropy degree with increasing confinement is not definite either. As confining pressure increases, the anisotropic strength ratio (ASD) always decreases, while the anisotropic strength difference (ASD) increases or decreases depending on the friction strength of the rock block and anisotropic structural plane;
(5) The different anisotropic structural plane types may lead to distinct behaviors under high confinement, i.e., soft-filled layers or hard contact discontinuities-induced anisotropy.

This research presents an elaborate analysis on confinement-affected strength anisotropy by distinguishing the soft and hard anisotropic structures and extends the knowledge on such a fundamental topic in rock mass geomechanics. The analysis in this study has some tolerable limitations using the linear Mohr–Coulomb strength criterion to depict the strength of rock block and anisotropic structural plane under a wide range of confining pressures. Despite this, the theoretical analysis agrees well with the experiments, and the new understanding in this study can provide guidance for anisotropic rock mass engineering in high geostress environments. The directionally controlled progressive failure and the corresponding engineering measures of anisotropic rock mass are of concern and need further research urgently.

## Figures and Tables

**Table 1:** Strength parameters of anisotropic rock mass.

Rock Type	$c_{rb}$ (MPa)	$\varphi_{rb}$ (°)	$c_{as}$ (MPa)	$\varphi_{as}$ (°)	$c_{rb}\cot\varphi_{rb}$ (Mpa)	$c_{as}\cot\varphi_{as}$ (Mpa)	Variation with Increasing Confinement		
							Possibility of Anisotropic Structural Plane-Controlled Strength	Anisotropic Strength Ratio	Anisotropic Strength Difference
Dolomitic limestone	78.23	34.5	63.56	27	113.8	124.8	Increase	Decrease	Increase
Sandstone	26.92	47.92	16.45	33.5	25.57	24.93	Decrease	Decrease	Increase
Jointed plaster of Paris	3.67	21.26	0.86	32.56	9.30	1.35	Decrease	Decrease	Decrease
